# MeLSI: Metric Learning for Statistical Inference in Microbiome Community Composition Analysis

**DOI:** 10.64898/2025.12.04.692328

**Published:** 2025-12-04

**Authors:** Nathan Bresette, Aaron C. Ericsson, Carter Woods, Ai-Ling Lin

## Abstract

Microbiome beta diversity analysis relies on distance-based methods including PERMANOVA combined with fixed ecological distance metrics (Bray-Curtis, Euclidean, Jaccard, and UniFrac), which treat all microbial taxa uniformly regardless of their biological relevance to community differences. This “one-size-fits-all” approach may miss subtle but biologically meaningful patterns in complex microbiome data. We present MeLSI (Metric Learning for Statistical Inference), a novel machine learning framework that learns data-adaptive distance metrics optimized for detecting community composition differences in multivariate microbiome analyses. MeLSI employs an ensemble of weak learners using bootstrap sampling, feature subsampling, and gradient-based optimization to learn optimal feature weights, combined with rigorous permutation testing for statistical inference. The learned metrics can be used with PERMANOVA for hypothesis testing and with Principal Coordinates Analysis (PCoA) for ordination visualization. Comprehensive validation on synthetic benchmarks and real datasets shows that MeLSI maintains proper Type I error control while delivering competitive or superior F-statistics when signal structure aligns with CLR-based weighting and, crucially, supplies interpretable feature-weight profiles that clarify which taxa drive group separation. On the Atlas1006 dataset, MeLSI achieved stronger effect sizes than the best traditional methods, and even when performance was comparable, the learned feature weights provided biological insight that fixed metrics cannot supply. MeLSI therefore offers a statistically rigorous tool that augments beta diversity analysis with transparent, data-driven interpretability.

**IMPORTANCE:**

Understanding which microbes differ between groups of interest could reveal therapeutic targets and diagnostic biomarkers. However, current analysis methods treat all microbes equally (similar to using the same ruler to measure everything, regardless of what matters most). This means subtle but clinically important differences may go undetected, especially when only a few key species drive disease while hundreds of “bystander” species add noise. MeLSI solves this by learning which microbes matter most for each specific comparison. In comparing male and female gut microbiomes, MeLSI identified specific bacterial families driving the differences, providing actionable biological insights that standard methods miss. This capability is particularly crucial for detecting early disease biomarkers, where differences are subtle and masked by biological variability. By telling researchers not just whether groups differ, but which specific microbes drive those differences, MeLSI accelerates the path from microbiome data to testable biological hypotheses and clinical applications.

## Full Text Availability

The license terms selected by the author(s) for this preprint version do not permit archiving in PMC. The full text is available from the preprint server.

